# Supplementary material for: Hybrid Models and Biological Model Reduction with PyDSTool
Source: PLoS Comput Biol. 2012 Aug 9;8(8):e1002628. doi: 10.1371/journal.pcbi.1002628 (PMC3415397; doi:10.1371/journal.pcbi.1002628)
Supplement: Text S4 — Complete source code for the PyDSTool package (version 0.88.120504). Includes API documentation and help files linking to web pages. This file is identical to the current public release on Sourceforge.net. (ZIP) [file pcbi.1002628.s004.zip › PyDSTool/html/PyDSTool.PyCont.misc-pysrc.html]

xml version="1.0" encoding="ascii"?


PyDSTool.PyCont.misc


| Home | Trees | Indices | Help | PyDSTool |
| --- | --- | --- | --- | --- |

Package PyDSTool ::
Package PyCont ::
Module misc

[hide private]

[frames] | no frames]

# Source Code for Module PyDSTool.PyCont.misc

```
  1  """ Common functions
 
  2  
 
  3      Drew LaMar, March 2006
 
  4  """ 
  5  
 
  6  # ----------------------------------------------------------------------------
 
  7  # Version History:
 
  8  #   February 2007
 
  9  #       Works with SciPy 0.5.2
 
 10  #
 
 11  #   September 2006
 
 12  #       Modified todict() to not update and return entire parsdict.  NOT COMPLETELY
 
 13  #           SURE OF EFFECT THIS WILL CREATE!
 
 14  #
 
 15  #   April 2006
 
 16  #       Added partition() function used in display() for plotting stability info
 
 17  #
 
 18  #   March 2006
 
 19  #       Added unique() function to make a list of items remove duplicate elements
 
 20  #
 
 21  # ----------------------------------------------------------------------------
 
 22  
 
 23  from PyDSTool import pointsToPointset, Point, Pointset 
 24  from PyDSTool.common import args 
 25  from PyDSTool.matplotlib_import import * 
 26  
 
 27  # THESE ARE REPEATS FROM CONTINUATION!  MAKE SURE AND UPDATE!!!
 
 28  all_point_types = ['P', 'RG', 'LP', 'BP', 'H', 'BT', 'ZH', 'CP', 'GH',
 
 29                     'DH', 'LPC', 'PD', 'NS', 'MX', 'UZ'] 
 30  all_curve_types = ['EP', 'LP', 'H', 'FP', 'LC'] 
 31  
 
 32  from time import clock 
 33  from scipy import linalg 
 34  from numpy import dot as matrixmultiply 
 35  from numpy import array, float, complex, int, float64, complex64, int32, \
 
 36       zeros, divide, subtract, argmax, identity, argsort, conjugate, sqrt, \
 
 37       arange, Inf, NaN, isfinite, r_, c_, sign, mod, mat, sum, \
 
 38       multiply, transpose, eye, real, imag, ndarray 
 39  from math import pi as PI 
 40  from copy import copy 
 41  
 
 42  #####
 
 43  _classes = ['IterationError', 'Struct'] 
 44  
 
 45  _functions = ['iszero', 'isnotzero', 'todict', 'tocoords', 'jac', 'hess',
 
 46                'hess3', 'bilinearform', 'trilinearform', 'ijtoind', 'indtoij',
 
 47                'testindij', 'wedge', 'invwedge', 'bialttoeig',
 
 48                'getFlowMaps', 'getFlowJac', 'getLeftEvecs',
 
 49                'firstlyapunov', 'unique', 'partition', 'CheckHopf', 'monotone'] 
 50  
 
 51  __all__ = _classes + _functions 
 52  #####
 
 53  
 
 54  FLOQ_TOL = 0.01 
 55  
 


56 -class IterationError(Exception):


57      pass

 58      
 


59 -class Struct(object):


60 -    def __init__(self, **entries):


61          self.__dict__.update(entries)

 62      
 


63 -    def __repr__(self):


64          attributes = [attr for attr in dir(self) if attr[0] != '_'] 
 65          string = 'Struct(' + ', '.join(attributes) + ')' 
 66          return string

 67  
 
 68  iszero = lambda x, y: x*y < 0 
 69  isnotzero = lambda x, y: x*y > 0 
 70  
 


71 -def monotone(x, num=None, direc=1):


72      """Checks to see if the list 'x' is increasing/decreasing ('direc'
 
 73      = 1/-1, respectively). If 'num' is specified, then it checks the
 
 74      first 'num' indices in 'x' if num > 0 and the last 'num' indices
 
 75      in 'x' if num < 0.    
 
 76      """ 
 77      if num is None: 
 78          ind = range(len(x)-1) 
 79      elif num > 1: 
 80          ind = range(num-1) 
 81      elif num < -1: 
 82          ind = range(num, -1) 
 83      else: 
 84          raise 'Number of indices must be larger than 1.' 
 85          
 
 86      mon = True 
 87      for i in ind: 
 88          if direc*(x[i+1]-x[i]) < 0: 
 89              mon = False 
 90              break 
 91      
 
 92      return mon

 93      
 


94 -def todict(C, X):


95      # Variables
 
 96      VARS = dict(zip(C.varslist,array(X)[C.coords])) 
 97      
 
 98      # Free parameters
 
 99      for i, par in enumerate(C.freepars): 
100          VARS.update({par: X[C.params[i]]}) 
101      #for i, par in enumerate(C.freepars):
 
102      #   C.parsdict[par] = X[C.params[i]]
 
103      #VARS.update(C.parsdict)
 
104      
 
105      # Auxiliary parameters
 
106      for i, par in enumerate(C.auxpars): 
107          VARS.update({par: X[C.params[C.freeparsdim+i]]}) 
108      return VARS

109      
 


110 -def tocoords(C, D):


111      X = zeros(C.dim, float) 
112      for i in range(C.varsdim): 
113          X[i] = D[C.varslist[i]] 
114      for i in range(C.freeparsdim): 
115          X[C.varsdim + i] = D[C.freepars[i]] 
116      for i in range(C.auxparsdim): 
117          X[C.varsdim + C.freeparsdim + i] = D[C.auxpars[i]] 
118      return X

119  
 


120 -def jac(func, x0, ind):


121      """Compute (n-1) x m Jacobian of function func at x0, where n =
 
122      len(x0) and m = len(ind).  ind denotes the indices along which to
 
123      compute the Jacobian.
 
124  
 
125      NOTE:  This assumes func has 1 more variable than it has equations!!    
 
126      """ 
127      
 
128      eps = 1e-6 
129      n = len(x0) 
130      m = len(ind) 
131      J = zeros((n-1, m), float) 
132      for i in range(m): 
133          ei = zeros(n, float) 
134          ei[ind[i]] = 1.0 
135          J[:,i] = (func(x0+eps*ei)-func(x0-eps*ei))/(2*eps) 
136      return J

137      
 


138 -def hess(func, x0, ind):


139      """Computes second derivative using 2nd order centered finite difference scheme.""" 
140      eps = 1e-3 
141      n = len(x0) 
142      m = len(ind) 
143      H = zeros((func.m,m,m), float) 
144      for i in range(m): 
145          ei = zeros(n, float) 
146          ei[ind[i]] = 1.0 
147          for j in range(i,m): 
148              ej = zeros(n, float) 
149              ej[ind[j]] = 1.0 
150              if i == j: 
151                  H[:,i,j] = (-1*func(x0+2*eps*ei) + 16*func(x0+eps*ei) - \
 
152                              30*func(x0) + 16*func(x0-eps*ei) - \
 
153                              func(x0-2*eps*ei))/(12*eps*eps) 
154              else: 
155                  H[:,i,j] = H[:,j,i] = (func(x0+eps*(ei+ej)) - \
 
156                              func(x0+eps*(ei-ej)) - func(x0+eps*(ej-ei)) + \
 
157                              func(x0-eps*(ei+ej)))/(4*eps*eps) 
158      return H

159      
 


160 -def hess3(func, x0, ind):


161      """Computes third derivative using hess function.""" 
162      eps = sqrt(1e-3) 
163      n = len(x0) 
164      m = len(ind) 
165      C = zeros((func.m,m,m,m), float) 
166      for i in range(m): 
167          ei = zeros(n, float) 
168          ei[ind[i]] = 1.0 
169          C[i,:,:,:] = (hess(func,x0+eps*ei,ind) - hess(func,x0-eps*ei,ind))/(2*eps) 
170      return C

171      
 


172 -def bilinearform(A, x1, x2):


173      return matrixmultiply(transpose(x2),matrixmultiply(A,x1))

174      
 


175 -def trilinearform(A, x1, x2, x3):


176      dim = A.shape 
177      return matrixmultiply(transpose([bilinearform(A[i,:,:],x1,x2) \
 
178                                       for i in range(dim[0])]),x3)

179      
 


180 -def ijtoind(i, j):


181      """ 0 <= j < i """ 
182      return i*(i-1)/2 + j

183      
 


184 -def indtoij(ind):


185      #size = array([n*(n-1)/2 - k*(k-1)/2 for k in range(1,n+1)])
 
186      #temp = nonzero(size <= ind)[0]
 
187      #j = n - temp
 
188      #i = j+1 + ind - size[temp]
 
189      
 
190      x = 0.5*(1 + sqrt(1 + 8*ind)) 
191      i = int(x) 
192      j = int(round(i*(x-i))) 
193      
 
194      return i, j

195      
 


196 -def testindij(n):


197      bn = n*(n-1)/2 
198      print "Testing %d..." % n 
199      for ind in range(bn): 
200          i, j = indtoij(ind) 
201          ind2 = ijtoind(i, j) 
202          if ind != ind2: 
203              print "DAMNIT!\n"

204          #print "  %d ---> (%d,%d) ---> %d" % (ind,i,j,ind2)
 
205      
 


206 -def wedge(u, v):


207      n = u.shape[0] 
208      bn = n*(n-1)/2 
209      q = zeros((bn,1), float) 
210      
 
211      for ind in range(bn): 
212          i, j = indtoij(ind) 
213          q[ind] = u[j]*v[i] - u[i]*v[j] 
214      
 
215      return q

216      
 


217 -def invwedge(q, n):


218      ind = argmax(abs(q),0)[0] 
219      q = q/q[ind] 
220      
 
221      i, j = indtoij(ind) 
222      
 
223      v1 = zeros((n,1), float) 
224      v2 = zeros((n,1), float) 
225      
 
226      v1[i,0] = 0 
227      v2[j,0] = 0 
228      v1[j,0] = 1 
229      v2[i,0] = 1 
230      for k in range(0,i): 
231          if k != j: 
232              v1[k,0] = q[ijtoind(i, k),0] 
233      for k in range(i+1,n): 
234          v1[k,0] = -1*q[ijtoind(k, i),0] 
235      
 
236      for k in range(0,j): 
237          v2[k,0] = -1*q[ijtoind(j, k),0] 
238      for k in range(j+1,n): 
239          if k != i: 
240              v2[k,0] = q[ijtoind(k, j),0] 
241      
 
242      return v1, v2

243  
 


244 -def bialttoeig(q, p, n, A):


245      v1, v2 = invwedge(q, n) 
246      w1, w2 = invwedge(p, n) 
247      
 
248      A11 = bilinearform(A,v1,v1) 
249      A22 = bilinearform(A,v2,v2) 
250      A12 = bilinearform(A,v1,v2) 
251      A21 = bilinearform(A,v2,v1) 
252      v11 = matrixmultiply(transpose(v1),v1) 
253      v22 = matrixmultiply(transpose(v2),v2) 
254      v12 = matrixmultiply(transpose(v1),v2) 
255      D = v11*v22 - v12*v12 
256      k = (A11*A22 - A12*A21)/D 
257  
 
258      return k[0][0], v1, w1

259  
 


260 -def firstlyapunov(X, F, w, J_coords=None, V=None, W=None, p=None, q=None,
 
261                    check=False):


262      if J_coords is None: 
263          J_coords = F.jac(X, F.coords) 
264      
 
265      if p is None: 
266          alpha = bilinearform(transpose(J_coords),V[:,0],V[:,1]) - \
 
267                  1j*w*matrixmultiply(V[:,0],V[:,1]) 
268          beta = -1*bilinearform(transpose(J_coords),V[:,0],V[:,0]) + \
 
269                  1j*w*matrixmultiply(V[:,0],V[:,0]) 
270          q = alpha*V[:,0] + beta*V[:,1] 
271          
 
272          alpha = bilinearform(J_coords,W[:,0],W[:,1]) + \
 
273                  1j*w*matrixmultiply(W[:,0],W[:,1]) 
274          beta = -1*bilinearform(J_coords,W[:,0],W[:,0]) - \
 
275                  1j*w*matrixmultiply(W[:,0],W[:,0]) 
276          p = alpha*W[:,0] + beta*W[:,1] 
277          
 
278          p /= linalg.norm(p) 
279          q /= linalg.norm(q) 
280          
 
281          direc = conjugate(1/matrixmultiply(conjugate(p),q)) 
282          p = direc*p 
283      
 
284      if check: 
285          print 'Checking...' 
286          print '  |q| = %f' % linalg.norm(q) 
287          temp = matrixmultiply(conjugate(p),q) 
288          print '  |<p,q> - 1| = ', abs(temp-1)    
289          print '  |Aq - iwq| = %f' % linalg.norm(matrixmultiply(J_coords,q) - 1j*w*q) 
290          print '  |A*p + iwp| = %f\n' % linalg.norm(matrixmultiply(transpose(J_coords),p) + 1j*w*p) 
291      
 
292      # Compute first lyapunov coefficient
 
293      B = F.hess(X, F.coords, F.coords) 
294      D = hess3(F, X, F.coords) 
295      b1 = array([bilinearform(B[i,:,:], q, q) for i in range(B.shape[0])]) 
296      b2 = array([bilinearform(B[i,:,:], conjugate(q),
 
297                            linalg.solve(2*1j*w*eye(F.m) - J_coords, b1)) \
 
298                   for i in range(B.shape[0])]) 
299      b3 = array([bilinearform(B[i,:,:], q, conjugate(q)) \
 
300                   for i in range(B.shape[0])]) 
301      b4 = array([bilinearform(B[i,:,:], q, linalg.solve(J_coords, b3)) \
 
302                   for i in range(B.shape[0])]) 
303      temp = array([trilinearform(D[i,:,:,:],q,q,conjugate(q)) \
 
304                   for i in range(D.shape[0])]) + b2 - 2*b4 
305  
 
306      l1 = 0.5*real(matrixmultiply(conjugate(p), temp)) 
307      
 
308      return l1

309  
 


310 -def CheckHopf(C, X):


311      J_coords = C.CorrFunc.jac(X, C.coords) 
312      eigs = linalg.eig(J_coords,left=0,right=0) 
313      
 
314      # Check for neutral saddles
 
315      found = False 
316      for i in range(len(eigs)): 
317          if abs(real(eigs[i])) < 1e-5: 
318              for j in range(i+1, len(eigs)): 
319                  if abs(real(eigs[j])) < 1e-5 and \
 
320                     abs(real(eigs[i]) - real(eigs[j])) < 1e-5: 
321                      found = True 
322                      T = 2*PI/abs(imag(eigs[i])) 
323      
 
324      if found: 
325          return T 
326      else: 
327          return None

328  
 


329 -def unique(s):


330      """
 
331      Return a list of the elements in s, but without duplicates.
 
332  
 
333      For example, unique([1,2,3,1,2,3]) is some permutation of [1,2,3],
 
334      unique("abcabc") some permutation of ["a", "b", "c"], and
 
335      unique(([1, 2], [2, 3], [1, 2])) some permutation of
 
336      [[2, 3], [1, 2]].
 
337  
 
338      For best speed, all sequence elements should be hashable.  Then
 
339      unique() will usually work in linear time.
 
340  
 
341      If not possible, the sequence elements should enjoy a total
 
342      ordering, and if list(s).sort() doesn't raise TypeError it's
 
343      assumed that they do enjoy a total ordering.  Then unique() will
 
344      usually work in O(N*log2(N)) time.
 
345  
 
346      If that's not possible either, the sequence elements must support
 
347      equality-testing.  Then unique() will usually work in quadratic
 
348      time.
 
349  
 
350      Author: Tim Peters (2001/04/06)
 
351      """ 
352  
 
353      n = len(s) 
354      if n == 0: 
355          return [] 
356  
 
357      # Try using a dict first, as that's the fastest and will usually
 
358      # work.  If it doesn't work, it will usually fail quickly, so it
 
359      # usually doesn't cost much to *try* it.  It requires that all the
 
360      # sequence elements be hashable, and support equality comparison.
 
361      u = {} 
362      try: 
363          for x in s: 
364              u[x] = 1 
365      except TypeError: 
366          del u  # move on to the next method 
367      else: 
368          return u.keys() 
369  
 
370      # We can't hash all the elements.  Second fastest is to sort,
 
371      # which brings the equal elements together; then duplicates are
 
372      # easy to weed out in a single pass.
 
373      # NOTE:  Python's list.sort() was designed to be efficient in the
 
374      # presence of many duplicate elements.  This isn't true of all
 
375      # sort functions in all languages or libraries, so this approach
 
376      # is more effective in Python than it may be elsewhere.
 
377      try: 
378          t = list(s) 
379          t.sort() 
380      except TypeError: 
381          del t  # move on to the next method 
382      else: 
383          assert n > 0 
384          last = t[0] 
385          lasti = i = 1 
386          while i < n: 
387              if t[i] != last: 
388                  t[lasti] = last = t[i] 
389                  lasti += 1 
390              i += 1 
391          return t[:lasti] 
392  
 
393      # Brute force is all that's left.
 
394      u = [] 
395      for x in s: 
396          if x not in u: 
397              u.append(x) 
398      return u

399  
 


400 -def partition(a, elems):


401      """Current issue with neutral points changing past bifurcation
 
402      point. False advertising as well: Not really a general function
 
403      (specialized for my use)
 
404      """     
405      delems = {'X': []} 
406      for n in range(len(elems)): 
407          delems[elems[n]] = [] 
408          
 
409      loc1 = loc2 = 0 
410      elem = a[0] 
411      while loc2 < len(a): 
412          loc2 += 1 
413          if loc2 == len(a) or a[loc2] != elem: 
414              if a[loc2-1] == 'N': 
415                  delems[elem].append([loc1,loc2]) 
416                  loc1 = loc2-1 
417              else: 
418                  delems[elem].append([loc1,loc2+1]) 
419                  loc1 = loc2 
420  
 
421              if loc2 != len(a): 
422                  # Move loc2 to list of elems larger than 1 in length and save as unknown curve
 
423                  if a[loc2] != 'N': 
424                      while loc2 < len(a)-1 and a[loc2] != a[loc2+1]: 
425                          loc2 += 1 
426                      if loc2-loc1 > 1: 
427                          delems['X'].append([loc1,loc2+1]) 
428                          loc1 = loc2 
429                      
 
430                  elem = a[loc2] 
431  
 
432      return delems

433      
 


434 -def negate(x):


435      if isinstance(x, dict): 
436          for k, v in x.iteritems(): 
437              x[k] = -1*v 
438      elif isinstance(x, Point): 
439          for i in range(len(x)): 
440              x[i] = -1*x[i] 
441      elif isinstance(x, Pointset): 
442          for i in range(len(x)): 
443              for j in range(len(x[i])): 
444                  x[i][j] = -1*x[i][j] 
445      elif isinstance(x, ndarray): 
446          x = -1*x 
447      elif isinstance(x, args): 
448          for k, v in x.iteritems(): 
449              x[k] = negate(x[k]) 
450      else: 
451          raise TypeError("Invalid argument type given") 
452          
 
453      return x

454  
 
455  
 


456 -def getFlowJac(pt, verbose=False):


457      try: 
458          jac0 = pt.labels['LC']['data'].jac0 
459          jac1 = pt.labels['LC']['data'].jac1 
460      except AttributeError: 
461          raise RuntimeError("Malformed point -- no Jacobian information.") 
462  
 
463      J = linalg.solve(jac1, jac0) 
464      if verbose: 
465          print "Jacobian J*x" 
466          print "------------\n" 
467          print J 
468          print "\n" 
469          
 
470          print "Check Jacobian" 
471          print "--------------\n" 
472          print "   eigs = ", linalg.eig(J)[0] 
473          print "   eigs = ", pt.labels['LC']['data'].evals 
474  
 
475      return J

476  
 
477  
 


478 -def getFlowMaps(n, pt, pttype, method='standard'):


479      """
 
480      method: 'standard' is currently the only working method for calculating
 
481        the flow maps.
 
482      """ 
483  
 
484      try: 
485          flow = pt.labels[pttype]['flow']  # flow maps (matrices) 
486      except: 
487          raise RuntimeError("Malformed point -- no flow map information.") 
488      ntst = len(flow)/2 
489      maps = [] 
490      if method=='standard': 
491          for i in range(ntst): 
492              I = identity(n) 
493              for j in mod(arange(i, i + ntst), ntst): 
494                  j = int(j) 
495                  I = linalg.solve(flow[2*j+1], matrixmultiply(flow[2*j], I)) 
496              maps.append(I) 
497      else: 
498          raise RuntimeError('method %s not supported'%method) 
499      return maps, ntst

500  
 
501  
 


502 -def getLeftEvecs(n, ntst, maps, flow_vecs, method='standard', verbose=False):


503      """Get left eigenvetors w corresponding to the unit eigenvalue of flow,
 
504      normalized so that w.v = 1, where v are flow vectors.
 
505  
 
506      method: 'standard' is currently the only working method for calculating
 
507        the flow maps and left eigenvectors.
 
508      """ 
509      evals = [] 
510      levecs = [] 
511      revecs = []   # unused unless verbose (for debugging) 
512      for m in maps: 
513          w, vl, vr = linalg.eig(m, left=1, right=1) 
514          evals.append(w) 
515          levecs.append(vl) 
516          revecs.append(vr)  # unused unless verbose (for debugging) 
517      idxs = range(ntst) 
518  
 
519      if verbose: 
520          print "Eigenvalues:", [evals[i] for i in idxs] 
521          check1 = linalg.norm(matrixmultiply(maps[i], revecs[i][:,ind]) - \
 
522                          evals[i][ind]*revecs[i][:,ind]) 
523          check2 =  linalg.norm(matrixmultiply(transpose(levecs[i][:,ind]),
 
524                          maps[i]) - evals[i][ind]*transpose(levecs[i][:,ind])) 
525          if check1 > 1e-5 or check2 > 1e-5: 
526              raise RuntimeError("Bad eigenvectors of monodromy matrix") 
527      
 
528      if method == 'standard': 
529          # all left eigenvectors w are given in the array evec1
 
530          evec1 = evec1_standard(idxs, evals, levecs) 
531      else: 
532          raise RuntimeError('Method %s not supported'%method) 
533      
 
534      ### Normalization
 
535      # (flow vectors eval'd at limit cycle points, assuming mesh points are evenly spaced)
 
536      evn = multiply(evec1, flow_vecs) 
537      # Divide each column of evec1 through by the length of w*v
 
538      return evec1/array([sum(evn, 1)]*n).T

539  
 
540  
 
541  
 


542 -def evec1_standard(idxs, evals, levecs):


543      """Standard method"""         
544      evec1 = [] 
545      for i in idxs: 
546          ind = argsort(abs(evals[i]-1.))[0] 
547          if abs(evals[i][ind]-1) > FLOQ_TOL: 
548              raise RuntimeError("Bad Floquet multipliers") 
549          
 
550          vsgn = 1 
551          if i > 1: 
552              vsgn = sign(matrixmultiply(transpose(evec1[-1]),
 
553                                         levecs[i][:,ind])) 
554          evec1.append(vsgn*levecs[i][:,ind]) 
555      return array(evec1)

556
```

  


| Home | Trees | Indices | Help | PyDSTool |
| --- | --- | --- | --- | --- |

|  |  |
| --- | --- |
| Generated by Epydoc 3.0.1 on Fri May 4 15:24:18 2012 | http://epydoc.sourceforge.net |
